# Functional genomics identifies negative regulatory nodes controlling phagocyte oxidative burst

**DOI:** 10.1038/ncomms8838

**Published:** 2015-07-21

**Authors:** Daniel B. Graham, Christine E. Becker, Aivi Doan, Gautam Goel, Eduardo J. Villablanca, Dan Knights, Amanda Mok, Aylwin C.Y. Ng, John G. Doench, David E. Root, Clary B. Clish, Ramnik J. Xavier

**Affiliations:** 1 Broad Institute of MIT and Harvard, Cambridge, Massachusetts 02142, USA; 2 Department of Medicine, Massachusetts General Hospital, Harvard Medical School, Boston, Massachusetts 02114, USA; 3 Center for Computational and Integrative Biology, Massachusetts General Hospital, Harvard Medical School, Boston, Massachusetts 02114, USA; 4 Department of Computer Science and Engineering, University of Minnesota, Minneapolis, Minnesota 55455, USA; 5 Gastrointestinal Unit and Center for the Study of Inflammatory Bowel Disease, Massachusetts General Hospital, Harvard Medical School, Boston, Massachusetts 02114, USA; 6 Center for Microbiome Informatics and Therapeutics, Massachusetts Institute of Technology, Cambridge, Massachusetts 02139, USA

## Abstract

The phagocyte oxidative burst, mediated by Nox2 NADPH oxidase-derived reactive oxygen species, confers host defense against a broad spectrum of bacterial and fungal pathogens. Loss-of-function mutations that impair function of the Nox2 complex result in a life-threatening immunodeficiency, and genetic variants of Nox2 subunits have been implicated in pathogenesis of inflammatory bowel disease (IBD). Thus, alterations in the oxidative burst can profoundly impact host defense, yet little is known about regulatory mechanisms that fine-tune this response. Here we report the discovery of regulatory nodes controlling oxidative burst by functional screening of genes within loci linked to human inflammatory disease. Implementing a multi-omics approach, we define transcriptional, metabolic and ubiquitin-cycling nodes controlled by *Rbpj, Pfkl* and *Rnf145*, respectively. Furthermore, we implicate Rnf145 in proteostasis of the Nox2 complex by endoplasmic reticulum-associated degradation. Consequently, ablation of *Rnf145* in murine macrophages enhances bacterial clearance, and rescues the oxidative burst defects associated with *Ncf4* haploinsufficiency.

## Abstract (web summary)

Phagocytes employ multiple bactericidal mechanisms to kill microorganisms, including the generation of toxic superoxide and other reactive oxygen species. Here the authors utilize a multi-omics approach to identify and characterize new regulatory nodes implicated in mucosal immunity that control phagocyte oxidative burst.

## Graphical abstract

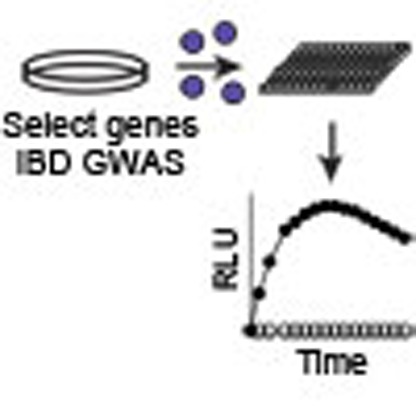

Phagocytes such as neutrophils, macrophages and dendritic cells deploy multiple bactericidal mechanisms to kill microorganisms^1^. During the earliest stages of pathogen encounter, phagocytes generate toxic superoxide and other reactive oxygen species (ROS) in phagosomes to kill microbes by oxidation of DNA, lipids and iron–sulfur centres within critical metabolic enzymes^2^. Oxidative burst requires assembly of the Nox2 complex on the phagosomal membrane, which subsequently catalyses the conversion of di-atomic oxygen into superoxide radicals by utilizing NADPH as a cofactor for electron transfer^3^,^4^. Recruitment of Nox2 NADPH oxidase regulatory subunits (p40^phox^, p47^phox^ and p67^phox^) from the cytosol to the membrane-associated catalytic subunits (gp91^phox^ and p22^phox^) requires signalling from receptors such as integrins, G-protein-coupled receptors or C-type lectins specialized in detection of pathogens and inflammatory mediators.

Many loss-of-function alleles have been described for Nox2 NADPH oxidase subunits, with x-linked *CYBB* (gp91^phox^) being the most common cause of chronic granulomatous disease (CGD), a life-threatening primary immunodeficiency associated with recurrent bacterial and fungal infections^5^. More rare autosomal recessive forms of CGD have been attributed to loss-of-function mutations in additional Nox2 complex subunits such as *NCF1* (p47^phox^), *NCF2* (p67^phox^) and *CYBA* (p22^phox^)^5^. In one reported case, autosomal recessive inheritance of two *NCF4* (p40^phox^) null alleles was associated with a unique clinical profile relative to other forms of CGD and manifested as severe colitis resembling IBD^6^. In addition to the observation that all forms of CGD can be associated with inflammatory gastrointestinal disease, the notion that immunodeficiency can lead to pathological inflammation driven by commensal microorganisms is also supported by the recent discovery of hypomorphic alleles of Nox2 complex subunits linked to IBD^6^,^7^,^8^.

Over several decades, a great deal of mechanistic insight into regulation of the Nox2 complex has been gained from a combination of characterizing cases of primary immunodeficiency in humans and in the study of knockout mice. As a result of these loss-of-function studies, many required genes have been discovered in the oxidative burst pathway, but few negative regulators have been identified. Among pathways that positively regulate oxidative burst, adhesion-dependent signalling through immunoreceptor tyrosine-based activation motifs (ITAMs) profoundly augments Nox2 NADPH oxidase activity in response to inflammatory mediators^9^. In this context, Src and Syk kinases direct the assembly of a signalling complex comprised of Slp76, Vav and PLC-γ2, which elicits calcium flux and production of diacylglycerol. Signal amplification from these second messengers promotes PKC-mediated phosphorylation of Nox2 complex regulatory subunits^10^,^11^, while Vav guanine nucleotide exchange factors activate Rac GTPases for catalytic induction of NADPH oxidase activity^12^,^13^. The prevailing strategy for identifying signalling mediators controlling oxidative burst has been a candidate-based approach in which selection of candidates requires some knowledge of their function. Given that much of the genome is incompletely annotated at functional resolution, it has remained a challenge to discover novel regulatory nodes, especially negative regulators, within signalling pathways. Here we describe a strategy for unbiased candidate selection and functional screening to identify regulators of oxidative burst.

With the rapid advancement of genomic technology, it is now possible to associate genetic variation with immune phenotypes at the population level. In particular, genome-wide association studies (GWAS) have implicated genetic loci associated with risk for IBD and allowed for inference of new biological processes that contribute to disease^14^. These studies highlight innate defense mechanisms such as antibacterial autophagy, superoxide generation during oxidative burst and reactive nitrogen species produced by iNOS^14^,^15^. However, a wealth of information from GWAS is untapped and will require functional analysis to unlock new insights. For example, many risk loci are densely populated with coding genes, which complicates identification of causal genes. Even when fine mapping clearly identifies key genes, a majority have poorly defined functions in host immunity. Moreover, any given gene may have multiple functions depending on the cell type in which it is expressed as well as environmental cues. Such context-specific functions of immune regulatory genes are largely unexplored. Thus, human genetics offers an opportunity to leverage insight from large amounts of genetic variation within healthy and patient populations to interrogate mechanisms of immunity. Irrespective of their putative roles in IBD pathology, genes within risk loci are likely to be highly enriched for genes controlling immune signalling pathways. Here we identify three novel negative regulatory nodes within the oxidative burst pathway, by functional screening of candidates implicated in disorders of inflammation. We further define the mechanistic basis of transcriptional, metabolic and ubiquitin-dependent regulation of the oxidative burst.

## Results

### Functional genetic screen for phagocyte oxidative burst

**Figure 1:**
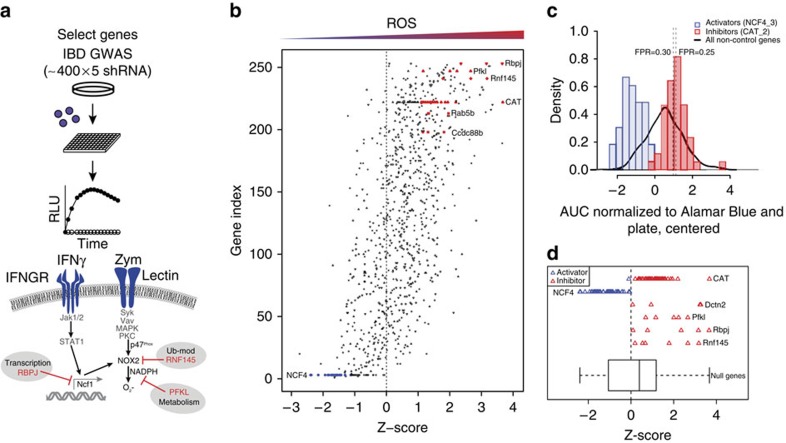
Screening GWAS genes identifies critical negative regulatory nodes controlling macrophage oxidative burst. (**a**) Workflow of primary screen. Genes associated with IBD and autoimmunity were screened to identify regulatory nodes controlling oxidative burst in RAW264.7 macrophages. Gene knockdown was achieved by lentiviral transduction with shRNAs, and cells were stimulated with IFN-γ and zymosan to induce oxidative burst as measured kinetically by luminol chemiluminescence. (**b**) For each hairpin tested, oxidative burst was quantified as the area under the curve (AUC) derived from kinetic luminescence plots, which was normalized to cell number (Alamar Blue) and ultimately expressed as *Z*-scores. (**c**) Among >400 genes screened, hits were classified as either activators or inhibitors of oxidative burst by using *Ncf4* and *Cat* as phenotypic references. FPR, false positive rate. (**d**) We identified four novel negative regulators (inhibitors) of the oxidative burst with robust false positive rates. See also Supplementary Data 1.

Accumulating evidence demonstrates the importance of phagocyte oxidative burst in maintaining host defense and immune homeostasis in mucosal tissues. Specifically, coding variants in *NCF4* and *NCF2* are associated with IBD^15^. Thus we reasoned that impaired oxidative burst mediated by the Nox2 complex represents a central pathway in mucosal immunity. Furthermore, we postulated that IBD GWAS loci would contain previously unknown regulators of the Nox2 NADPH oxidase complex. To identify novel regulatory nodes controlling oxidative burst, we performed a functional knockdown screen of genes present within IBD loci, those associated with Mendelian inheritance of IBD, and select genes involved in inflammatory pathways associated with autoimmunity. In total, we screened >400 candidate genes for functional effects on oxidative burst in RAW264.7 macrophages (Fig. 1a,b). Knockdown of candidate genes and controls (*Ncf4* and *Cat*) was achieved by lentiviral delivery of shRNAs with an average coverage of 5 hairpins per gene, for a total of ∼2,000 unique hairpins screened individually. After lentiviral delivery of shRNAs and selection, RAW cells were stimulated with zymosan yeast particles to engage dectin-1-dependent ITAM signalling and induce NADPH oxidase activation. Kinetics of ROS production was monitored over the course of 1 h with the chemiluminescent substrate luminol. In parallel, duplicate samples were assessed for cell viability by Alamar Blue and target gene knockdown by quantitative PCR (qPCR). Results from the primary screen demonstrate the sensitivity of the assay and ability to identify *Ncf4* as a positive regulator and *Cat* as a negative regulator. Furthermore, known ROS activators such as *Card9* scored as positive regulators in the screen^16^. To identify negative regulatory nodes in the ROS pathway, we devised a hit-calling strategy using a structured Bayesian approach to control for the per gene false discovery rate (FDR; Fig. 1c). This approach incorporated the experimental structure into the FDR estimation to share strength among multiple shRNAs for the same gene, thus allowing genes with low but consistent support across shRNAs to be identified. A key strength of this approach is in integration of multiple data measurements from multiple hairpins per gene to minimize false positive calls due to off-target effects and/or inefficient target gene knockdown (Supplementary Data 1). Accordingly, we used *Ncf4* to define the parameters of the activator class and *Cat* for the inhibitor class. *Cat* was selected during assay development as a top performing control, presumably due to its effect on cellular hydrogen peroxide levels and/or peroxidase activity. By defining activator and inhibitor classes based on controls (Fig. 1c), we prioritized genes for further investigation and focused on three novel genes that negatively regulated phagocyte oxidative burst, each representing a putative regulatory node (Fig. 1d). We identified Dctn2 as a negative regulator, suggesting that perhaps vesicular trafficking and/or phagolysosomal fusion terminate Dectin-1 signalling. In addition, we identified a transcriptional node controlled by Rbpj, a Notch-dependent transcription factor; a metabolic node controlled by Pfkl, the rate-limiting enzyme in glycolysis, and a ubiquitin-cycling node controlled by Rnf145, a multipass transmembrane protein with a C-terminal RING domain.

**Figure 2:**
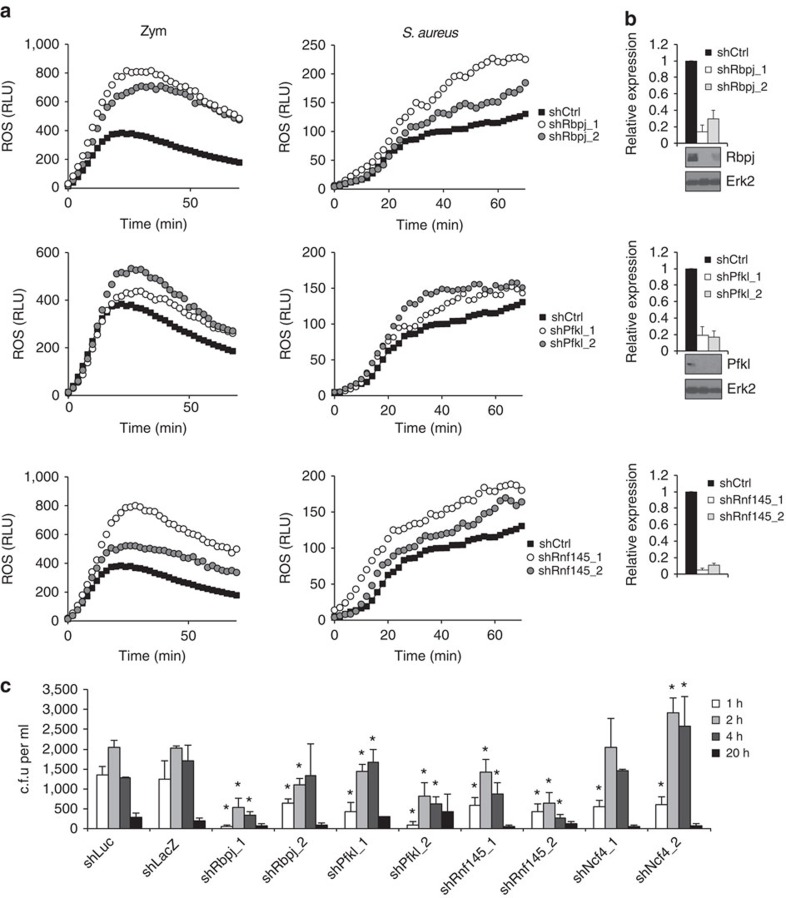
Targeting negative regulatory nodes controlling oxidative burst enhances bactericidal activity in macrophages. (**a**) Hits from the primary screen were validated and functionally characterized using the top two hairpins per gene, as defined by target gene knockdown efficiency in the primary screen (see Supplementary Data 1). Accordingly, RAW264.7 cells were subjected to shRNA knockdown followed by IFN-γ priming and zymosan or *Staphylococcus aureus* stimulation to induce oxidative burst. Data represent ROS production as relative luminescence units (RLU) measured with luminol substrate. (**b**) Target gene knockdown was measured by qPCR and normalized to *Actb* housekeeping gene or by western blot. Data represent mean±s.d. of biological triplicates. (**c**) The indicated genes were knocked down by shRNA in RAW264.7 cells. After IFN-γ priming, macrophages were infected with *Staphylococcus aureus* for 30 min, washed and cultured in gentamicin media to kill any bacteria that had not been phagocytosed. After the indicated time points, cells were lysed, and remaining live bacteria were enumerated as colony-forming units (c.f.u.). Data represent the mean of triplicates±s.d. *P≤0.05 as determined by Student's *t*-test.

Based on primary screening results, we proceeded with further functional characterization of hits. Accordingly, we selected two hairpins per gene based on knockdown efficiency and confirmed that ablation of Rbpj, Pfkl or Rnf145 resulted in augmented oxidative burst in response to zymosan or *Staphylococcus aureus* (Fig. 2a,b). To test the significance of increased ROS production on host defense, we monitored bactericidal activity of macrophages after perturbation of candidate negative regulatory nodes. We chose *S. aureus* for these experiments, as this species is sensitive to superoxide and is frequently associated with chronic infection in CGD^5^. After knockdown of candidate genes, macrophages were cultured with bacteria for 30 min to allow for phagocytosis. After washing away free bacteria, macrophages were cultured in the presence of gentamicin to kill any remaining extracellular bacteria. Bacterial killing was monitored by lysing macrophages and determining the number of colony-forming units (c.f.u.) of remaining live bacteria. As a control, *Ncf4* knockdown resulted in impaired bacterial killing, as evidenced by increased bacterial burden (c.f.u. at 2 h; Fig. 2c). Consistent with augmented ROS production, knockdown of *Rbpj*, *Pfkl* and *Rnf145* resulted in more effective bacterial killing relative to control shRNAs (Fig. 2c). Thus, perturbation of negative regulatory nodes within the ROS pathway can enhance bacterial killing.

**Figure 3:**
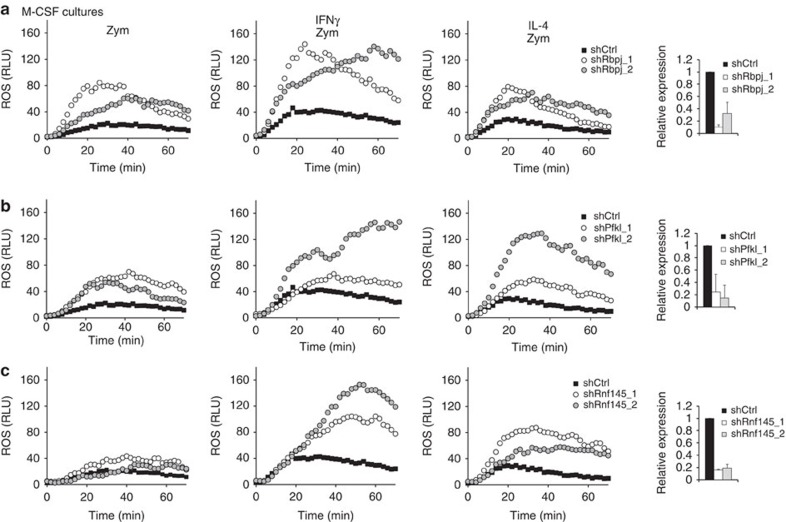
Targeting negative regulatory nodes in primary macrophages enhances oxidative burst. Primary murine bone marrow-derived macrophages were transduced with shRNA targeting *Rbpj* (**a**), *Pfkl* (**b**), *Rnf145* (**c**), or controls targeting luciferase or LacZ. Cells were left unprimed or primed with either IFN-γ or IL-4 prior to stimulation with zymosan to induce oxidative burst. Data represent ROS production as relative luminescence units (RLU) measured with luminol substrate. Target gene knockdown was measured by qPCR and normalized to *Actb* housekeeping gene. Data represent mean±s.d. of biological triplicates.

Having identified three negative regulatory nodes in the oxidative burst response in RAW cells, we sought to determine if these nodes are functional in primary bone marrow-derived macrophages (BMDMs) and if this is affected by distinct priming stimuli. Thus we knocked down candidate genes in BMDMs and measured the oxidative burst response to zymosan in unprimed cells or those that had been primed with IFN-γ or IL-4. Our results indicate that Rbpj and Pfkl function as negative regulators of zymosan-induced oxidative burst regardless of the priming stimuli being tested (Fig. 3a,b). In contrast, Rnf145 represented a negative regulatory node on priming with IFN-γ or IL-4 but not in unprimed cells (Fig. 3c). We next addressed the possibility that these three regulatory nodes function in a cell type-dependent manner by performing the same experiment in bone marrow-derived dendritic cells (BMDCs). Although *Rbpj* and *Pfkl* knockdown trend towards enhanced zymosan-induced oxidative burst in primed BMDCs, *Rnf145* knockdown did not (Supplementary Fig. 1). Thus, the oxidative burst may be controlled by distinct regulatory nodes in different cell types and under different stimulation conditions. To further dissect molecular regulation of these nodes in macrophages, we systematically evaluated Rbpj in the context of transcription, Pfkl in metabolism and then focused predominantly on Rnf145 because of its novel role in ubiquitin-dependent control of Nox2 function.

### Rbpj is a transcriptional node in the oxidative burst

**Figure 4:**
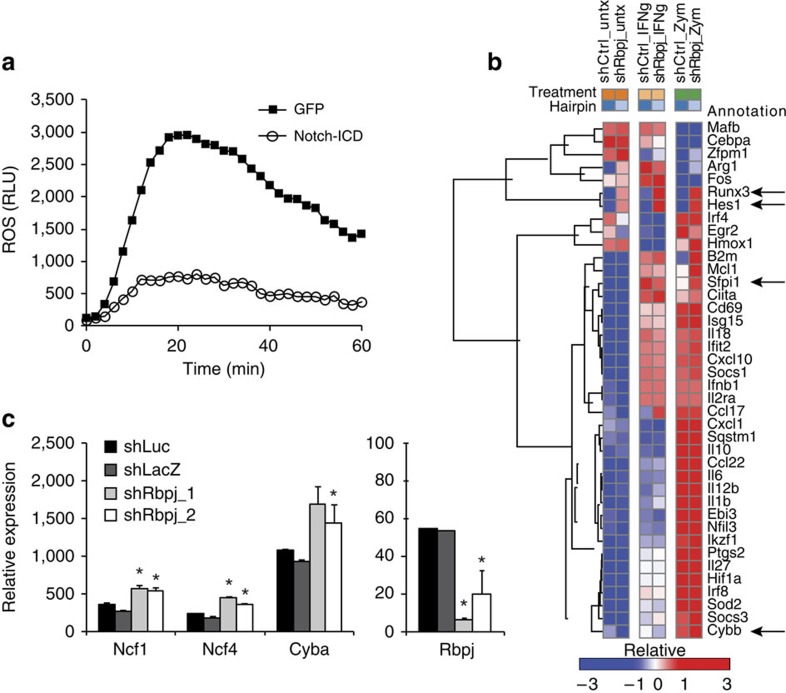
Rbpj negatively regulates a myeloid transcriptional programme controlling expression of *Ncf1* (p47^phox^). (**a**) RAW264.7 cells were transduced with lentivirus encoding constitutively active Notch1 ICD expressed from bicistronic IRES–GFP or IRES–GFP alone. Zymosan-induced oxidative burst was measured by luminol chemiluminescence as in Fig. 2a–c. Primary bone marrow-derived macrophages were transduced with lentivirus encoding the indicated shRNAs. After 6 h of treatment with the indicated stimuli, macrophages were lysed and subjected to transcriptional profiling by Fluidigm (**b**) and qPCR (**c**). Data represent the mean of triplicates±s.d. *P≤0.05 as determined by Student's *t*-test.

Enhanced ROS production and antibacterial activity after *Rbpj* knockdown suggests Notch regulation in macrophages, and we thus sought to determine if Notch signalling directly impacts ROS production in macrophages. In support of this model, ectopic expression of the constitutively active Notch1 intracellular domain (Notch-ICD) inhibited oxidative burst, suggesting that Notch is a negative regulator in this context (Fig. 4a). To identify transcriptional programmes controlled by the Notch–Rbpj axis in myeloid cells, we knocked down *Rbpj* in BMDMs and performed transcriptional profiling. Towards this end, we designed a custom Fluidigm panel to measure expression of cytokines, inflammatory markers and transcription factors (Supplementary Data 2). Strikingly, *Rbpj* knockdown led to increased expression of the Notch target *Hes1* (Fig. 4b). Given that Rbpj acts as a transcriptional repressor in the absence of Notch signalling and an activator in its presence, our results suggest that intrinsic Notch signalling is absent or low in cultured macrophages. Thus, Rbpj largely functions as a repressor in this context. Indeed, *Rbpj* knockdown dramatically upregulated the transcription factor *Runx3* and the master regulator of gp91^phox^ expression in myeloid cells *Sfpi1* (PU.1)^17^ (Fig. 4b). Together, Runx3 and PU.1 function cooperatively to induce expression of myeloid-specific genes^18^. Consistent with this notion, our transcriptional profiling further demonstrated an upregulation (∼20%) of gp91^phox^ (*Cybb*) on knockdown of *Rbpj*. In addition, *Rbpj* knockdown in BMDMs resulted in enhanced expression of *Ncf1, Ncf4, Cyba* and *Sfpi1* (Fig. 4b,c). Thus, our results demonstrate that repressive activity of Rbpj in macrophages inhibits a myeloid transcriptional programme associated with expression of Nox2 subunits.

### Pfkl regulates a metabolic node in the oxidative burst

**Figure 5:**
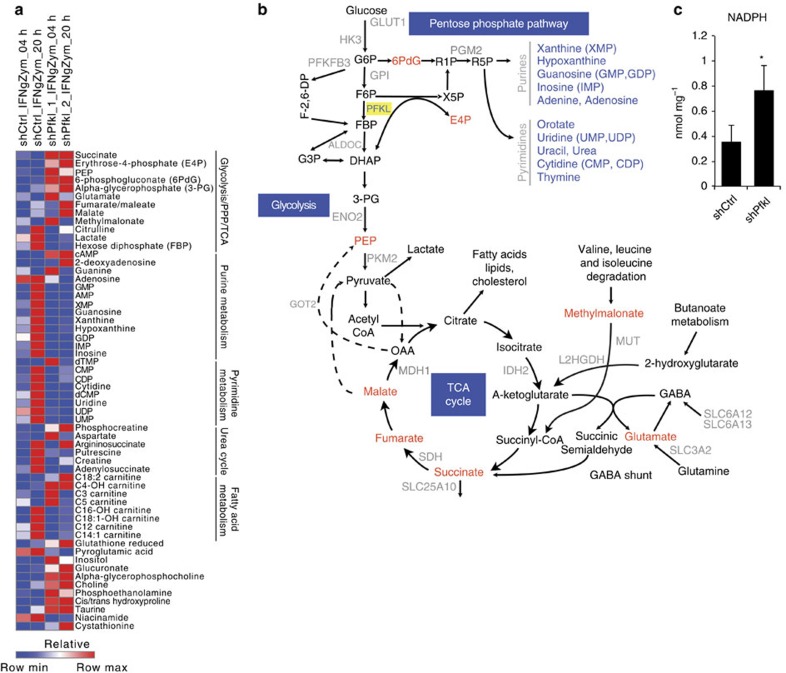
*Pfkl* knockdown impairs glycolysis and enhances flux through the pentose phosphate pathway. (**a**) Primary bone marrow-derived macrophages were transduced with lentivirus encoding the indicated shRNAs. At the indicated time point after stimulation with IFN-γ and zymosan, macrophages were lysed and subjected to metabolomic profiling. (**b**) Pathway activity map illustrating increased flux through the pentose phosphate pathway on *Pfkl* knockdown. Red text indicates elevated, and blue text indicates reduced metabolite on *Pfkl* knockdown. (**c**) RAW264.7 cells were transduced with the indicated shRNAs, and NADPH levels were quantified by enzymatic cycling assay. Data represent the mean of triplicates±s.d. *P≤0.05 as determined by Student's *t*-test.

To specifically delineate the role of Pfkl in the oxidative burst response, we tested the hypothesis that Pfkl controls a regulatory node functioning at the level of metabolism. Given that Pfkl is the rate-limiting enzyme in glycolysis, we determined the metabolic consequences of *Pfkl* knockdown in BMDMs stimulated over the course of 20 h with IFN-γ and zymosan. We evaluated the levels of ∼200 polar metabolites extracted from macrophages and quantified by mass spectrometry (MS). Consistent with the notion that phagocyte activation is associated with a metabolic switch from mitochondrial oxidative phosphorylation to glycolysis^19^,^20^, we observed increased lactate and decreased TCA intermediates (isocitrate) in control macrophages. However, *Pfkl* knockdown reduced glycolytic flux as evidenced by diminished lactate (Fig. 5a and Supplementary Data 3). Moreover, the bottleneck in glycolysis imposed by *Pfkl* knockdown resulted in diversion of glucose into the pentose phosphate pathway (PPP). In this context, we observed accumulation of early PPP intermediates (6-phosphogluconate and erythrose-4-phosphate) and depletion of late-stage metabolites (purines and pyrimidines) (Fig. 5a,b), which is consistent with increased flux through the pathway to compensate for impaired glycolysis and to meet the energy demands of the cell. Importantly, PPP is the major cellular source of NADPH, which functions as a critical cofactor for NADPH oxidase by providing reducing equivalents to generate superoxide radicals. Indeed, *Pfkl* knockdown resulted in an increase in intracellular NADPH relative to control knockdown (Fig. 5c). Thus, Pfkl controls a metabolic regulatory node that modulates oxidative burst by fine-tuning NADPH levels within macrophages. Consistent with our findings in macrophages, Pfkb has been shown to control glycolytic flux in T cells^21^. In T cells derived from patients with rheumatoid arthritis, Pfkfb3 expression was reduced, glycolysis was impaired and PPP flux was increased, resulting in impaired energy generation and senescence^21^. Thus, inhibition of glycolysis shunts glucose metabolism towards the PPP and increases cellular levels of NADPH, which functions as a cofactor for NADPH oxidase catalysis and superoxide production.

### Rnf145 is a ubiquitin-cycling node in the oxidative burst

**Figure 6:**
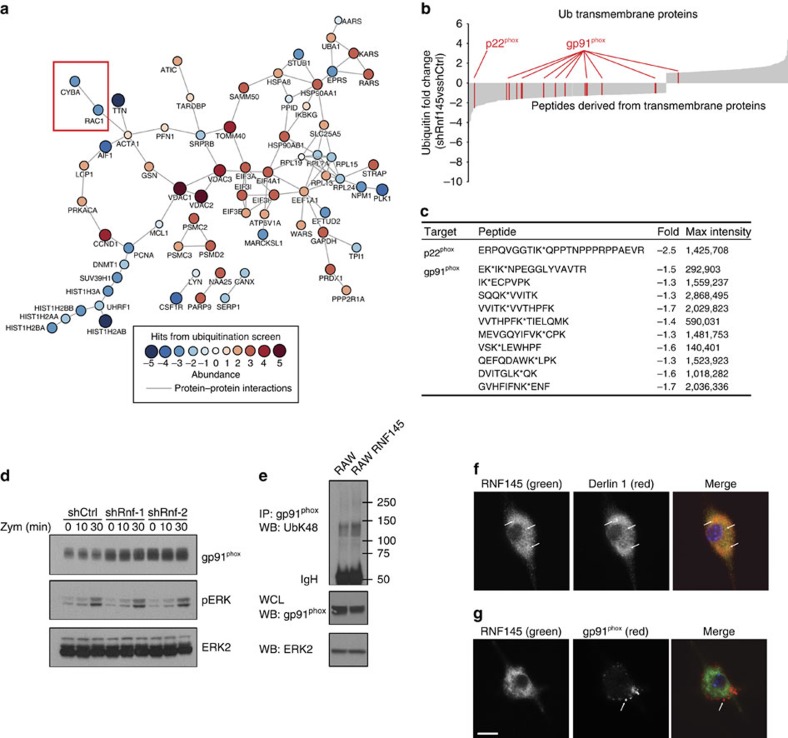
*Rnf145* knockdown reduces ubiquitination of the Nox2 complex. (**a**) RAW264.7 cells were transduced with lentivirus-encoding shRNA targeting Rnf145 or control (LacZ). Cells were then left untreated or stimulated with zymosan for 30 min. Cells were then lysed, and ubiquitinated peptides were immunoprecipitated and enumerated by UbiScan (see methods). Differential abundance of each ubiquitinated peptide was compared between control and *Rnf145* knockdown. The network was constructed by anchoring on hit components (>2.5-fold difference between *Rnf145* knockdown and control). Hits are represented by nodes and the network was extended by iteratively incorporating interacting partners using protein–protein interaction data derived from the curated Human Protein Reference Database and genome-wide interactome screens. The network was then pruned to include only first-order interactions (represented by edges) that directly connect the hits from UbiScan (enrichment *P*=2 × 10^−11^). (**b**) Relative abundance of ubiquitinated peptides derived from transmembrane proteins. Values represent fold difference between *Rnf145* knockdown and control. (**c**) Peptide sequences derived from p22^phox^ and gp91^phox^ showing ubiquitination sites (*), fold difference in *Rnf145* knockdown relative to control and relative abundance (max intensity). (**d**) Knockdown of *Rnf145* was performed in RAW264.7 macrophages (as in Fig. 2a), followed by stimulation with zymosan and western blot for the indicated proteins. (**e**) Rnf145 was expressed in RAW264.7 cells by lentiviral transduction. Cells were then lysed and analysed by western blot for gp91^phox^ and ERK2 as a loading control. In addition, gp91^phox^ was immunoprecipitated to detect K48-specific ubiquitin linkages by western blot. (**f**,**g**) RAW264.7 cells were transduced with lentivirus encoding Rnf145 with a C-terminal V5 epitope tag. Confocal microscopy was performed to visualize cellular localization of Rnf145 (V5), gp91^phox^ or Derlin 1. Scale bar, 10 μm. Arrows indicate colocalization of RNF145 and Derlin 1 (**f**) or gp91phox-positive vesicles (**g**).

To identify the function of Rnf145 in the oxidative burst response, we tested the hypothesis that Rnf145 controls a regulatory node functioning in the ubiquitin cycle. Given that Rnf145 contains a C-terminal RING domain and shares structural homology with several transmembrane-anchored E3 ubiquitin ligases involved in endoplasmic reticulum-associated degradation (ERAD), we sought to determine the effects of Rnf145 depletion on the ubiquitin landscape in macrophages. Towards this end, we knocked down *Rnf145* in RAW cells and compared the ubiquitin proteome to that in control cells with and without stimulation by IFN-γ and zymosan. After treatment, cells were lysed and subjected to trypsin digest prior to immunoprecipitation with a monoclonal antibody specific for the ubiquitin remnant (KGG) (Lys-Gly-Gly) present on trypsin-cleaved ubiquitin substrates. Ubiquitin substrates were then identified and quantified by MS. We observed several thousand ubiquitinated peptides in macrophages and ∼100 of these increased in abundance after zymosan stimulation. Among these were Irak2 and Ikk (Supplementary Data 4). However, given that Rnf145 is a transmembrane protein, we next compared ubiquitin levels in all transmembrane proteins from RAW cells with and without *Rnf145* knockdown. Strikingly, ubiquitinated peptides derived from gp91^phox^ and p22^phox^ were significantly less abundant in cells with *Rnf145* knockdown (Fig. 6a,b). Moreover, 11 distinct peptides from these Nox2 complex subunits were differentially ubiquitinated after *Rnf145* knockdown, and we defined specific lysine residues that were ubiquitinated (Fig. 6b,c). Notably, zymosan stimulation did not significantly alter ubiquitin levels of Nox2 complex subunits, suggesting that it is not likely to be ubiquitinated in phagosomes.

To determine if steady-state levels of the Nox2 complex are controlled by Rnf145 and ubiquitination, we depleted Rnf145 in RAW cells and observed elevated levels of gp91^phox^ as measured by western blot (Fig. 6d and Supplementary Fig. 1a). Importantly, zymosan stimulation did not appear to alter steady-state levels of gp91^phox^, suggesting that the role of Rnf145 in regulating Nox2 complex levels is independent of stimulation. Consistent with this notion, depletion of Rnf145 had no effect on zymosan-induced signalling events such as Erk phosphorylation (Fig. 6d), which is a requisite event for activation of the oxidative burst. Having shown increased levels of gp91^phox^ after *Rnf145* knockdown, we performed the converse experiment by overexpressing Rnf145 in RAW cells and measuring gp91^phox^ expression by western blot. Indeed, Rnf145 expression reduced protein levels of gp91^phox^ compared with the mock-transduced RAW cells (Fig. 6e). Moreover, we observed increased ubiquitin K48 linkages on gp91^phox^ after ectopic expression of Rnf145 (Fig. 6e and Supplementary Fig. 1b), suggesting that these conjugates are destined for proteosomal destruction through ERAD. In fact, the close paralogue of Rnf145, AMFR, has been shown to participate in ERAD^22^. Similarly, we observed that Rnf145 localizes to the ER along with the core ERAD protein Derlin 1 (Fig. 6f). While Rnf145 localized to the ER, we found that gp91^phox^ was widely distributed throughout the cell, with its primary address in vesicles and the plasma membrane (Fig. 6g). Thus, these data indicate that Rnf145 does not colocalize with mature gp91^phox^ in vesicular structures, but instead may interact with nascent gp91^phox^ protein translated on the rough ER.

**Figure 7:**
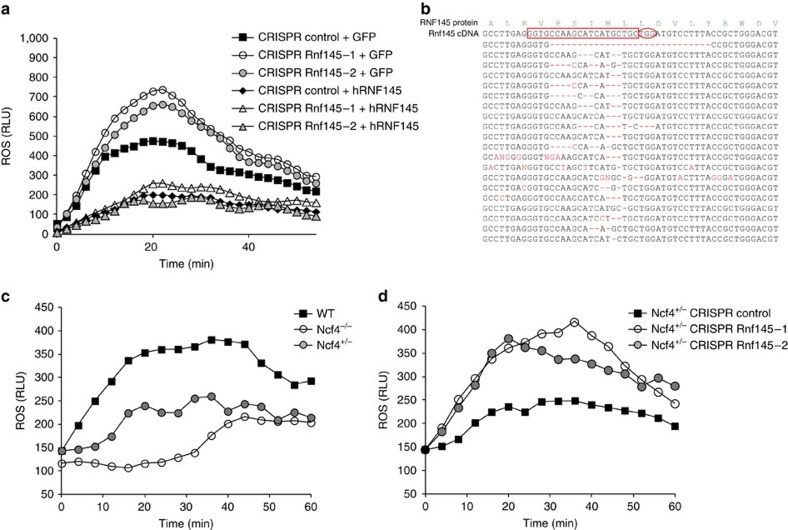
Rnf145 ablation in mouse macrophages rescues oxidative burst in *Ncf4*^+/−^ cells. (**a**) RAW264.7 cells were transduced with lentivirus-encoding Cas9 and guide RNA targeting murine *Rnf145* (or intergenic control). Where indicated, complementary DNA rescue was performed with human *RNF145* (or GFP control). Oxidative burst was monitored by luminol chemiluminescence. (**b**) Examples of indel mutations obtained from primary bone marrow-derived macrophages transduced with lentivirus-encoding Cas9 and guide RNA targeting Rnf145. The guide sequence is outlined in red and the PAM sequence is circled. (**c**) Oxidative burst in bone marrow-derived macrophages from the indicated mouse strains. (**d**) Oxidative burst in bone marrow-derived macrophages from *Ncf4*^+/−^ mice transduced with CRISPRs targeting *Rnf145*.

Having identified novel regulatory nodes in the oxidative burst pathway, we hypothesized that perturbation of these nodes could compensate for genetic lesions associated with IBD. We first deleted *Rnf145* in RAW cells using RNA-guided Cas9 nuclease^23^,^24^. Two independent CRISPR lines, each containing an indel mutation rate of ∼80% within the *Rnf145* coding sequence, led to augmented oxidative burst induced by zymosan (Fig. 7a). As a specificity control, we demonstrated that ectopic expression of human Rnf145 (but not green fluorescent protein (GFP)) in murine RAW cells reduced elevated ROS production in CRISPR-targeted cells (Fig. 7a). We then employed CRISPR technology in primary BMDMs (Fig. 7b) to determine if perturbation of the Rnf145 node can rescue defective oxidative burst associated with *Ncf4* deficiency. Like in human subjects, deletion of *Ncf4* in mice resulted in a severe impairment of Nox2 NADPH oxidase-derived phagosomal ROS (Fig. 7c). Moreover, haploinsufficiency of *Ncf4*, which may more accurately simulate IBD-associated *Ncf4* variants, led to a partial defect in oxidative burst (Fig. 7c). We reasoned that *Ncf4* haploinsufficiency decreases NADPH oxidase activity by altering the stoichiometry of regulatory subunits (p40^phox^ and indirectly p67^phox^) and decreases efficiency of their assembly. We hypothesized that increasing gp91^phox^ expression by CRISPR targeting of *Rnf145* may promote assembly of the complex even if the regulatory subunits are present at suboptimal levels. Strikingly, we found that CRISPR-mediated deletion of *Rnf145* in *Ncf4*^+/−^ macrophages rescued oxidative burst and restored the response to levels akin to wild type (Fig. 7c,d). Taken together, we leverage functional genomics to discover regulatory nodes controlling oxidative burst and show that perturbation at these nodes can bypass or compensate for genetic lesions associated with IBD.

## Discussion

Here we demonstrate the utility of human genetics as a source of candidate genes controlling critical regulatory nodes in immune pathways. Given genetic and functional evidence implicating phagocyte oxidative burst in mucosal immunity, we performed a functional genetic screen of genes within IBD risk loci. We based this strategy on the notion that IBD candidates will be enriched for regulators of key immune pathways, irrespective of their putative relationship with IBD. Thus, our objectives were threefold: (1) to assign function to incompletely annotated genes, (2) to identify critical regulatory nodes constraining oxidative burst and (3) to define the mechanistic basis of Nox2 NADPH oxidase regulation. While the connection between genetic variation and IBD pathophysiology is complex, our data are consistent with the notion that alterations in oxidative burst (either positive or negative) can impose selective pressure on microbial communities in the gut, and that this can precipitate dysbiosis resulting in maladaptive inflammatory pathology.

Transcriptional regulation of oxidative burst was first described with the discovery that IFN-γ primes phagocytes to massively enhance antibacterial function and oxidative burst^25^,^26^. Further investigation then revealed the mechanistic basis of Stat1-dependent transcriptional activation of Nox2 subunits^27^,^28^. In fact these studies motivated clinical trials demonstrating benefit among CGD patients from IFN-γ therapy, although, as it turns out, efficacy was independent of effects on Nox2 subunit expression^26^. While Nox2 NADPH oxidase transcriptional regulation is potently induced by IFN-γ and Stat1, we report that Rbpj acts conversely to negatively regulate expression of the Nox2 complex components *Ncf1*, *Ncf4*, *Cybb* and *Cyba* and their master regulator *Sfpi1* (PU.1). Depending on the transcriptional cofactors with which it interacts, Rbpj functions as a transcriptional activator or repressor downstream of Notch. Although Notch functions as an early lineage commitment factor in T lymphocyte development, it shapes myeloid differentiation in more nuanced ways. Mice congenitally deficient in nicastrin, a nonredundant Notch-activating protease, develop a malignant myeloproliferative disease dominated by cells expressing elevated levels of transcription factors that control myeloid-specific genes such as *Ncf1*, *cFms* and *Fcrg*^29^. Thus, Notch appears to constrain the myeloid compartment by repressing late-stage differentiation factors controlling phagocyte effector function. In fact, Notch2 is required to enforce late-stage differentiation of CD11b^+^ mucosal dendritic cells and CD8^+^ splenic dendritic cells^30^. Moreover, Notch signalling influences myeloid effector function by converging with Toll-like receptor pathways. In this context, intrinsic Notch signalling in macrophages restrains cytokine production in an Rbpj-dependent manner^31^. Our results suggest that intrinsic Notch signalling is low in cultured macrophages, as evidenced by the observation that *Rbpj* knockdown enhanced expression of the Notch target Hes1, and that Rbpj is known to function as a repressor in the absence of Notch signalling^32^. Of note, we also found that *Rbpj* knockdown leads to increased expression of *Runx3*, which has been shown by ChIP-seq to physically bind the *Cybb* promoter^33^. Given that Runx3 can function together with PU.1 to promote transcriptional activation of myeloid-specific genes in dendritic cells^18^, our data are consistent with a model in which Rbpj-mediated suppression of Runx3 limits PU.1-dependent transcription of Nox2 subunits. While the complexity of Notch–Rbpj transcriptional regulation in macrophages will require further investigation, our findings collectively highlight the role of Rbpj in restraining the oxidative burst by repressing expression of Nox2 complex subunits.

Post-transcriptional regulation of the Nox2 complex occurs at multiple levels. After assembly of the cytosolic regulatory subunits with membrane-associated gp91^phox^ and p22^phox^, catalytic activity of NADPH oxidase commences. Superoxide generation involves a two-step electron transfer reaction through cofactors bound to gp91^phox^. First, gp91^phox^ binds NADPH, which donates an electron to FAD. In turn, FAD transfers an electron to haem and subsequently to molecular oxygen for production of superoxide radicals^3^. Thus, metabolic regulation of cellular redox state and NADPH levels form a restriction point that controls the catalytic rate of the oxidative burst. Our metabolomic analyses reveal that the rate-limiting glycolytic enzyme Pfkl regulates NADPH oxidase activity by affecting the PPP and NADPH levels. In the absence of Pfkl, glucose is diverted towards the PPP, which along with controlling purine metabolism is the primary source for cellular NADPH. We observed increased flux through the PPP and augmented oxidative burst in macrophages lacking Pfkl. Consistent with this notion, phagocyte activation by pathogen-associated molecular patterns and inflammatory mediators massively upregulates glucose uptake and utilization^34^. Furthermore, these activation pathways promote a metabolic switch from glycolysis to the PPP by recruiting phosphoglycerate mutase to the Nox2 complex^35^. Our findings identify a novel link between NADPH oxidase activation and the glycolytic enzyme Pfkl, which controls NADPH production through the PPP. Thus, metabolic adaptations induced by pathogen exposure reinforce innate effector functions such as oxidative burst.

In addition to identifying mechanisms controlling oxidative burst at the level of Nox2 subunit transcription (Rbpj) and catalytic function (Pfkl), we identify Rnf145 as a novel E3 ubiquitin ligase that regulates gp91^phox^ steady-state protein levels. This is the only E3 ligase that scored among more than a dozen that we screened. Very little is known about regulation and turnover of proteins within the Nox2 complex. Recent work identified gp91^phox^ as a caspase 1 substrate that is cleaved after inflammasome activation; however, this was an inducible event that controlled the duration and amplitude of oxidative burst and had no effect on baseline proteostasis^36^. More recently, gp91^phox^ protein levels were shown to be regulated by Lrrc33, although a specific ubiquitin ligase targeting the Nox2 complex was not identified^37^. Here we identify Rnf145 as required for ubiquitination of the Nox2 complex and precisely define ubiquitinated lysine residues in gp91^phox^ and p22^phox^. Our data support a model in which Rnf145 controls steady-state levels of the Nox2 complex by ubiquitination. Importantly, the function of Rnf145 as a central ubiquitin ligase controlling oxidative burst has relevant implications for host defense. In fact, perturbation of this ubiquitin-cycling node by ablation of Rnf145 in *Ncf4*-haploinsufficient macrophages restores oxidative burst to levels in *Ncf4*-replete cells. This example of epistatic compensation highlights the potential value of identifying regulatory nodes as therapeutic targets.

Taken together, we report key insights from mining genes within GWAS candidate loci and performing genetic screens followed by deep functional analysis. In particular, this approach has proven fruitful in defining central regulatory nodes in the oxidative burst pathway.

## Methods

### Primary screen and ROS assay

The primary screen was performed in murine RAW264.7 cells (ATCC) that were seeded at a density of 1 × 10^4^ per 100 μl per well in 96-well, clear bottom, black wall plates. After 24 h, each well was transduced with 20 μl of shRNA lentivirus (The RNAi Consortium, Broad Institute) supplemented with 8 μg ml^−1^ polybrene (Sigma) and centrifuged at 1,150*g* 25 °C for 30 min. The following day, media was exchanged and supplemented with 5 μg ml^−1^ puromycin (InvivoGen). Three days after selection, cell viability was approximated by Alamar Blue assay according to the manufacturer's recommendation (Life Technologies). Cells were primed overnight with 10 ng ml^−1^ (50 U ml^−1^) murine IFNγ (Peprotech) prior to assaying oxidative burst. Media was exchanged for assay buffer (PBS, 0.9 mM CaCl_2_, 0.5 mM MgCl_2_, 20 mM dextrose, 200 μM luminol, 25 U ml^−1^ horseradish peroxidase and 100 μg ml^−1^ zymosan or heat killed *S. aureus*) and plates were briefly centrifuged to settle zymosan particles. Luminol-derived chemiluminescence was then monitored kinetically in a BioTek SynergyH4 microplate reader every 3 min for 30 min with temperature control at 37 °C. Duplicate plates were designated for RNA extraction and validation of knockdown efficiency by qPCR. Where indicated, knockdown validation was performed by western blot with rabbit anti-Pfkl (AV45774, Sigma, dilution 1:1,000) or rabbit anti-Rbpj (5313S, Cell Signaling Technology, dilution 1:1,000). Where indicated, RAW264.7 cells were transduced with lentivirus encoding murine Rnf145 (C-terminal V5 epitope tag), GFP or human NOTCH1 ICD within bicistronic IRES–GFP cassette of the lentiviral backbone CSGW.

### Hit calling

The method assumes that each gene belongs to one of three classes (‘activator,' ‘null' or ‘inhibitor'), with the area under the ROS luminescence time series curve (area under the curve (AUC)) of each modelled as a Gaussian distribution of AUC values. We calculated the expected mean of the activator class using activator controls (*Ncf4*) from all plates, and that of the inhibitor class using all inhibitor controls (*Cat*) from all plates. To derive the expected mean of the null class, we took the mean of all non-control shRNAs that were not outliers using the boxplot rule (greater than 1.5 times the interquartile range below/above the first/third quartiles). We estimated the variance of each class using the pooled variance of the control genes. We then computed the normal likelihood of each shRNA in each class; the probability of gene membership in each class was then calculated as the product of the posterior probabilities that all constituent shRNAs for that gene belonged to that class. We then controlled the per gene FDR using the Benjamini–Hochberg procedure^38^. Before performing this analysis, we preprocessed the data as follows: (1) drop those shRNAs with low virus titre (<7.5 percentile in virus titre distribution); (2) drop those with low Alamar Blue score (<1,000); (3) drop those shRNAs with validated knockdown rates of <0.5; (4) normalize AUC to Alamar Blue score; (5) standardize by median/median absolute deviation within plate to control for plate effects; (6) centre data on zero based on positive control inhibitors and activators (for visualization purposes).

### Primary macrophage assays

Murine BMDMs were seeded at 3.5 × 10^4^ cells per 50 μl per well in 96-well, clear bottom, black wall plates. Following 24–48 h in culture with granulocyte-macrophage colony-stimulating factor, cells were transduced with 50 μl of the indicated lentiviral shRNA and cultured for an additional 3 days, at which point media was supplemented with puromycin at a concentration of 4 μg ml^−1^. After 2 days in selection, cells were primed with IFN-γ (25 ng ml^−1^) or IL-4 (25 ng ml^−1^) and stimulated with zymosan as described for the RAW264.7 screen.

### Bacterial killing assay

RAW264.7 cells were plated at 5 × 10^4^ per well in 96-well plates in DMEM with 10% FBS overnight. *S. aureus* (Reynolds strain capsular serotype 5) was grown overnight at 37 °C in Columbia media supplemented with 2% NaCl and sub-cultured the next day to the mid-exponential phase (OD_600_=0.8−0.9). One hour before infection, media was replaced with DMEM with 1% FCS. *S. aureus* bacterial particles were disrupted by passing 10 to 15 times through a 30-G needle with a 1-ml syringe and were then added at a multiplicity of infection of 5. The plate was centrifuged for 5 min at 515*g*, 4 °C and incubated at 37 °C for 30 min to allow internalization of *S. aureus* by macrophages. Cells were washed three times with 200 μl Dulbecco's phosphate-buffered saline (DPBS) to remove non-adherent cells and bacteria. 200 μl of fresh media containing gentamicin (final concentration of 300 μg ml^−1^) was added to kill extracellular bacteria. After 1 h of incubation, media was replaced with fresh DMEM containing 100 μg ml^−1^ gentamicin. At indicated time points (time after internalization), supernatants were removed and cells were washed three times in 200 μl DPBS. Cells were lysed in 200 μl of 0.02% Triton X-100, serial dilutions of the bacterial suspensions were plated on blood agar (Remel) and colonies were counted after overnight incubation at 37 °C.

### Transcriptional readouts

Knockdown of the indicated target genes in murine BMDMs was achieved as described above. Following selection and stimulation, total RNA was extracted (Qiagen RNeasy Mini kit) and subjected to transcriptional profiling with the BioMark HD system (Fluidigm). In brief, purified total RNA was reverse transcribed (qScript Supermix, VWR) prior to target-specific pre-amplification by standard PCR. Custom primers (Supplementary Data 2) specific for inflammatory genes were utilized for pre-amplification followed by qPCR (EvaGreen Supermix, Bio-Rad).

### Metabolomics

Endogenous, polar metabolite profiles were measured using two separate hydrophilic interaction liquid chromatography (HILIC) tandem MS methods^39^. Polar metabolites were profiled in the positive ion MS mode using a liquid chromatography (LC) system comprised of a 1,200 Series pump (Agilent Technologies) and an HTS PAL autosampler (Leap Technologies) that was coupled to a 4,000 QTRAP mass spectrometer (AB SCIEX) equipped with an electrospray ionization source. Samples were prepared by drying 100 μl of cell extract under nitrogen and resuspending the residue in 100 μl of 10/67.4/22.4/0.2 v/v/v/v water/acetonitrile/methanol/formic acid containing stable isotope-labelled internal standards (valine-d8, Sigma-Aldrich; and phenylalanine-d8, Cambridge Isotope Laboratories). The samples were centrifuged (10 min, 10,000 r.p.m., 4 °C) and the supernatants were injected directly onto a 150 × 2.1 mm Atlantis HILIC column (Waters Corp.). The column was eluted isocratically with 5% mobile phase A (10 mM ammonium formate/0.1% formic acid) for 1 min followed by a linear gradient to 40% mobile phase B (acetonitrile/0.1% formic acid) over 10 min. The ion spray voltage was 4.5 kV and the source temperature was 425 °C. All metabolites were measured using multiple reaction monitoring scans. MS settings, including declustering potentials and collision energies, for each metabolite were optimized by infusion of reference standards prior to sample analyses.

Analyses of polar metabolites using negative ion mode MS were performed using an ACQUITY UPLC (Waters Corp.) coupled to a 5,500 QTRAP triple quadrupole mass spectrometer. Cell extracts in 80% methanol were directly injected onto a 150 × 2.0 mm Luna NH2 column (Phenomenex) that was eluted at a flow rate of 400 μl min^−1^ with initial conditions of 10% mobile phase A (20 mM ammonium acetate and 20 mM ammonium hydroxide in water) and 90% mobile phase B (10 mM ammonium hydroxide in 75:25 v/v acetonitrile/methanol) followed by a 10 min linear gradient to 100% mobile phase A. The ion spray voltage was −4.5 kV and the source temperature was 500 °C. All data were acquired using multiple reaction monitoring scanning.

MultiQuant software (version 2.1; AB SCIEX) was used to process all raw LC-MS data and integrate chromatographic peaks. The processed data were manually reviewed for quality of integration and compared against known standards to confirm metabolite identities.

Measurement of NADPH was performed by enzymatic cycling as described by the manufacturer (Abcam).

### Statistical analysis of metabolomics data

Fold-change levels in metabolites were analysed by a two-tailed *t*-test assuming equal variance between two samples. Metabolites with fold change >10% and Benjamin–Hochberg adjusted *P* value <0.01 were considered significant. Due to significant differences between wild-type and knockdown cells at baseline, each of the two knockdown samples were normalized to the two control samples independently and then assessed for differences in fold change. Differences at 4 h and 20 h were analysed separately. We selected metabolites that showed consistent signals across the two knockdown samples.

### Ubiquitin proteomics

*Rnf145* knockdown in RAW264.7 cells was performed as described above, with scale-up for 10 cm dishes. Cells were primed with IFN-γ (10 ng ml^−1^) overnight, and where indicated stimulated with zymosan (50 μg ml^−1^) for 30 min. Samples were processed at Cell Signaling Technology, Inc. using the UbiScan proteomic platform as previously described^40^,^41^,^42^,^43^,^44^,^45^. Samples were washed once with cold PBS and lysed in urea lysis buffer (20 mM HEPES pH 8.0, 9.0 M urea, 1 mM sodium orthovanadate, 2.5 mM sodium pyrophosphate, 1 mM β-glycerol-phosphate). Lysates were snap frozen on dry ice/ethanol until further processing. Cells were sonicated at 15 W output power twice for 20 s, and once for 15 s. The resulting sonicated lysates were centrifuged at 20,000*g* for 15 min to remove insoluble material. The yielded protein extracts were reduced and carboxamidomethylated.

After normalizing total protein for each cell line (15 mg of total protein each), proteins were digested overnight with trypsin (Worthington). Resulting peptides were separated from non-peptide material by solid-phase extraction with Sep-Pak Classic C18 cartridges (Waters Corp). Purified peptides were lyophilized for 3 days at 30 mtorr at −65 °C (Vertis). Lyophilized peptides were redissolved in IAP buffer (50 mM MOPS pH 7.2, 10 mM NaH_2_PO_4_, 50 mM NaCl), and ubiquitinated peptides were enriched by immunoaffinity purification using the Ubiquitin Remnant Motif antibody (#5562, Cell Signaling Technology) immobilized on Protein A agarose beads (Roche) following the standard protocol for immunoaffinity enrichment (http://www.cellsignal.com/services/pdfs/PTMScan_Proteomics_System_Protocol.pdf). Peptides were eluted from antibody-resin into a total volume of 100 μl in 0.15% trifluoroacetic acid (TFA), and concentrated with C18 STAGE tips^46^.

### Mass spectrometric analysis

The samples were run in duplicate to generate analytical replicates and increase the number of MS/MS identifications from each sample. Peptides were loaded directly onto a 10 cm × 75 μm PicoFrit capillary column packed with Magic C18 AQ reversed-phase resin. The column was developed with a 90-min linear gradient of acetonitrile in 0.125% formic acid delivered at 280 nl min^−1^. Tandem mass spectra were collected with an LTQ-Orbitrap VELOS hybrid mass spectrometer running XCalibur 2.0.7 SP1 using a top 20 method, a dynamic exclusion repeat count of 1 and a repeat duration of 30 s. Real-time recalibration of mass error was performed using lock mass with a singly charged polysiloxane ion (*m/z*=371.101237). MS spectra were collected in the Orbitrap component of the mass spectrometer, and MS/MS spectra were collected in the LTQ.

### Data analysis

Data processing was performed as described previously^47^. MS/MS spectra were processed using SEQUEST 3G and the SORCERER 2 platform from Sage-N Research (v4.0). Searches were performed against the *Mus musculus* NCBI database (6/28/2011). Reverse decoy databases were included for all searches to estimate false positive rates, and peptide assignments were obtained using a 5% false positive discovery rate in the Peptide Prophet module of SORCERER 2. Results were filtered using a mass accuracy of ±5 p.p.m. for precursor ions and 1 Da for product ions. Enzyme specificity was limited to trypsin, with at least one K or R terminus required per peptide and up to four mis-cleavages allowed. Cysteine carboxamidomethylation was specified as a static modification, and methionine oxidation and di-glycine (lysine epsilon-gly-gly modification) was allowed. Results were further narrowed to peptides containing the di-glycine modification. The final false positive discovery rate on motif-containing peptides ranged from 2.0 to 2.6%.

### Label-free quantitation

Label-free quantification was performed using Progenesis V4.1 (Nonlinear Dynamics) combined with previously described peak detection software^44^,^47^,^48^,^49^. MS1 peak intensities for ubiquitinated peptides identified in at least one sample were retrieved from the ion chromatogram files of all samples using a mass precision of ±5 p.p.m. and a retention time window of 5 min. Retention time warping (or chromatographic alignment) was performed across binary comparisons to allow retrieval of the correct peak intensity. Peak intensities for all peptides that changed in abundance between treatments were manually reviewed using XCalibur and the corresponding LC-MS files to ensure accuracy, and where necessary due to varying chromatographic peak shapes between samples peak height measurements were replaced with peak areas. Manually validated intensities are reported in Supplementary Data 4 in bold.

### Biochemistry

RAW264.7 cells were stimulated with zymosan (50 μg ml^−1^) for the indicated time points, washed in PBS and lysed (1% NP-40, 100 mM Tris, 100 mM NaCl, pH 8.0). Lysates were resolved by PAGE, transferred to Immobilon PVDF membranes (Millipore) and subjected to western blot. Primary antibodies were mouse anti-gp91^phox^ (611415, BD Pharmingen, dilution 1:1,000), mouse anti-pERK (9106S, Cell Signaling Technology, dilution 1:1,000) and rabbit anti-ERK2 (sc-154, Santa Cruz Biotechnology, dilution 1:2,000). Secondary antibodies were horseradish peroxidase conjugates of anti-mouse IgG and anti-rabbit IgG (7074S and 7076S, Cell Signaling Technology dilution, 1:5,000). For immunoprecipitation experiments, lysates were incubated overnight with protein A/G-coupled magnetic beads (Pierce) coupled to rabbit anti-gp91^phox^ (ab129068, Abcam, 5 μg per sample). Western blots were performed as described above with anti-Ubiquitin K48 (12805S, Cell Signaling Technology, dilution 1:1,000).

### Microscopy

IFN-γ-primed RAW264.7 cells were allowed to adhere to coverslips, then fixed in 4% paraformaldehyde for 15 min at 37 °C. Cells were then stained with mouse monoclonal anti-V5 (R960-25, Invitrogen dilution 1:250), polyclonal rabbit anti-gp91^phox^ (ab129068, dilution 1:250) and polyclonal rabbit anti-Derlin-1 (8897S, Cell Signaling Technology, dilution 1:250) diluted in staining buffer (3% BSA, 0.1% saponin, PBS). Secondary staining was performed with goat anti-rabbit IgG Alexa Fluor 488 (Invitrogen) and goat anti-mouse IgG Alexa Fluor 594 (Invitrogen). After thorough washing, cells were visualized using a laser-scanning confocal microscope (Leica SP5X) and analysed with ImageJ software (NIH).

### CRISPR

Guide RNAs targeting the mouse genome were selected from the CRISPR design tool and cloned into a pHKO lentiviral vector (a gift from B. Ebert, Harvard Medical School and Broad Institute)^50^. RAW264.7 or BMDMs were transduced with CRISPR lentivirus as described above, but with concentrated viral supernatant produced by centrifugal filtration (Millipore). *Ncf4*^*−/−*^ mice were a gift from P.T. Hawkins (Babraham Institute, Cambridge, UK).

### Animals

All animal studies were conducted under protocols approved by the Subcommittee on Research Animal Care (SRAC) at Massachusetts General Hospital. All mice have been backcrossed to the C57BL/6 background, and both sexes were utilized for bone marrow harvest at 8–12 weeks of age.

## Additional information

**How to cite this article:** Graham, D. B. *et al.* Functional genomics identifies negative regulatory nodes controlling phagocyte oxidative burst. *Nat. Commun.* 6:7838 doi: 10.1038/ncomms8838 (2015).

## Supplementary Material

Supplementary Figures Supplementary Figures 1-2

Supplementary Data 1 Primary ROS screen data. Oxidative burst response was measured kinetically by luminol chemiluminescence and expressed as area under the curve (ROS auc). Cell viability was determined by alamar blue fluorescence. Gene knockdown, as measured by qPCR, is expressed as percent expression relative to control (kdr).

Supplementary Data 2 Transcriptional profiling by Fluidigm. Primer sequences for Fluidigm assays.

Supplementary Data 3 Metabolite profiling. Identification and quantification of polar metabolites from LC/MS spectral data.

Supplementary Data 4 Ubiquitin profiling. Identification and quantification of ubiquitinated peptides from MS/MS spectral data.
