# Supplementary material for: Functional genomics identifies negative regulatory nodes controlling phagocyte oxidative burst
Source: Nat Commun. 2015 Jul 21;6:7838. doi: 10.1038/ncomms8838 (PMC4518307; doi:10.1038/ncomms8838)
Supplement: Supplementary Figures — 1-2 [file ncomms8838-s1.pdf]

Supplementary Figure 1

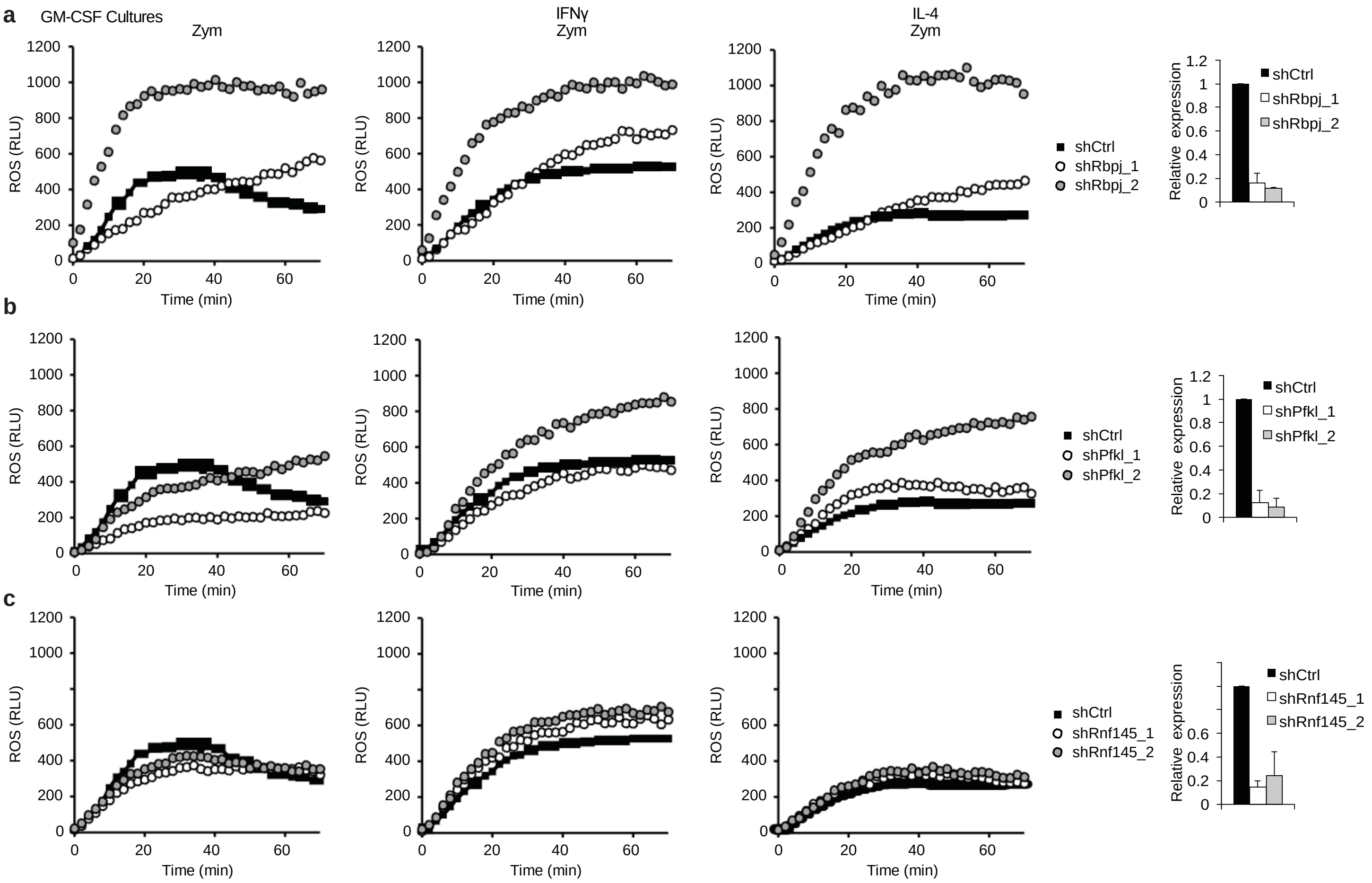

Supplementary Figure 1. Targeting negative regulatory nodes in primary dendritic cells enhances oxidative burst. Primary murine bone marrow derived dendritic cells were transduced with shRNA targeting Rbpj (a), Pfkl (b), Rnf145 (c), or controls targeting luciferase or LacZ. Cells were left unprimed or primed with either IFN-γ or IL-4 prior to stimulation with zymosan to induce oxidative burst. Data represent ROS production as relative luminescence units (RLU) measured with luminol substrate. Target gene knockdown was measured by qPCR and normalized to Actb housekeeping gene. Data represent mean ± s.d. of biological triplicates.

Supplementary Figure 2

a

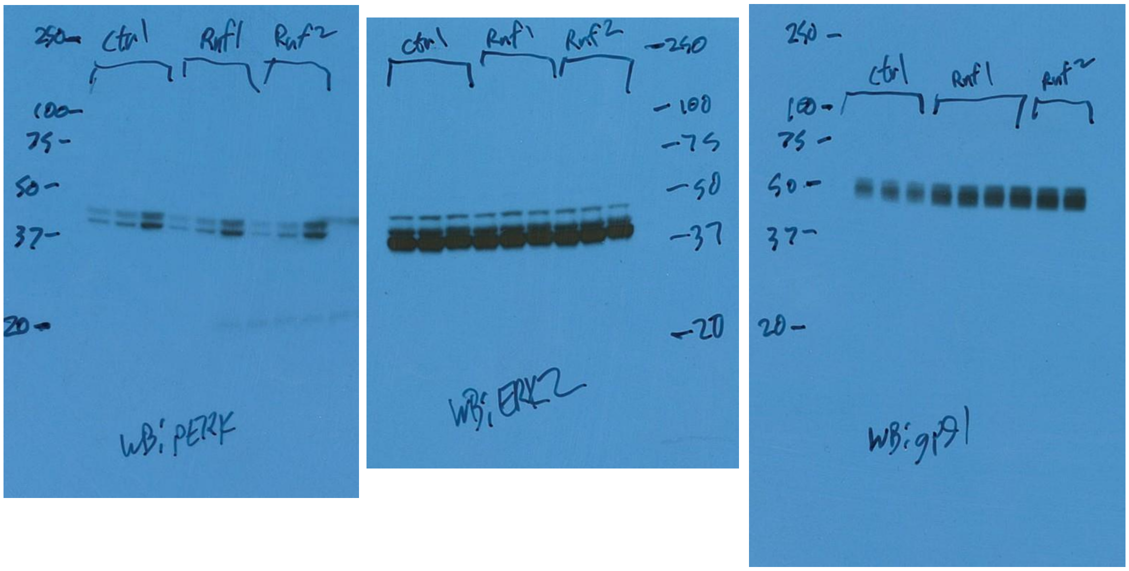

b

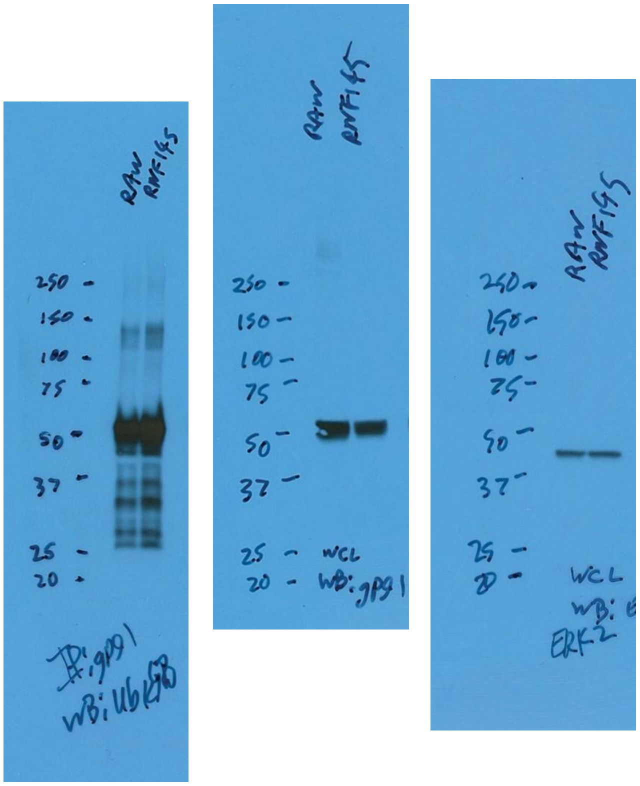

Supplementary Figure 2. Rnf145 regulates gp91phox expression. Supporting Figure 6. (a) Knockdown of Rnf145 was performed in RAW264.7 macrophages, followed by stimulation with zymosan, and Western blot for the indicated proteins. (b) Rnf145 was expressed in RAW264.7 cells by lentiviral transduction. Cells were then lysed and analyzed by western blot for gp91phox and ERK2 as a loading control. Additionally, gp91phox was immunoprecipitated to detect K48-specific ubiquitin linkages by western blot.
